# Astragaloside IV protects against iron loading‐induced abnormal differentiation of bone marrow mesenchymal stem cells (BMSCs)

**DOI:** 10.1002/2211-5463.13082

**Published:** 2021-03-17

**Authors:** Hui Jin, Jianyang Du, Huan Ren, Guofu Yang, Wenbo Wang, Jianyang Du

**Affiliations:** ^1^ Department of Orthopedics The First Affiliated Hospital of Harbin Medical University China; ^2^ Department of Neurosurgery The Second Affiliated Hospital of Harbin Medical University China

**Keywords:** astragaloside IV, BMSCs, dysfunction, iron homeostasis, iron loading, osteoporosis

## Abstract

Iron loading has been reported to be a common stress in the development of cells, and this might be related to bone loss and osteoporosis. Astragaloside IV (ASI‐IV), a pure compound derived from Radix Astragali, has been reported to exhibit cardioprotective, anti‐inflammatory, antioxidant, antiasthmatic and anticancer effects. The aim of this study was to investigate whether ASI‐IV could reverse iron loading‐induced inhibition of cell viability, proliferation, pluripotency and osteogenesis and promote adipogenesis of bone marrow mesenchymal stem cells (BMSCs). Ferric ammonium citrate (FAC) was used to stimulate iron loading conditions. ASI‐IV was observed to ameliorate the FAC‐induced reduction of cell viability, proliferation, pluripotency and osteogenesis of BMSCs. In addition, ASI‐IV could block the increased adipogenesis of BMSCs after FAC treatment. We intraperitoneally injected mice with 250 mg·kg^−1^ iron dextran, with or without ASI‐IV (40 mg·kg^−1^), for 4 weeks. ASI‐IV inhibited the iron loading‐induced bone loss of these mice. Furthermore, ASI‐IV played a protective role in iron loading‐induced abnormal differentiation of BMSCs by regulating iron homeostasis and metabolism. In summary, our study suggesteds that ASI‐IV might have potentials for development into a novel therapeutic strategy for the treatment of iron loading‐induced abnormal differentiation of BMSCs and osteoporosis.

## Graphical abstract

Ferric ammonium citrate (FAC)‐induced iron loading significantly inhibited the cell viability, proliferation, pluripotency and osteogenesis of bone marrow mesenchymal stem cells (BMSCs), but promoted their adipogenesis. Astragaloside IV (ASI‐IV) played a protective effect in iron loading‐induced abnormal viability, proliferation, pluripotency and differentiation of BMSCs via regulation of iron homeostasis and metabolism, mainly by regulating the expression of ferritin, transferrin receptor 1 and ferroportin 1.

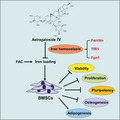

**Abbreviations**
ARS: Alizarin red S
ASI‐IV: astragaloside IV
BMD: bone mineral density
BMSC: bone marrow mesenchymal stem cell
Ca: calcium
CCK‐8: Cell Counting Kit‐8
CD: cluster of differentiation
DMEM: Dulbecco’s modified Eagle’s medium
DMT1: divalent metal ion binding protein
FAC: ferric ammonium citrate
Fe‐dextran: iron dextran
Mg: magnesium
micro‐CT: micro‐computed tomography
OP: osteoporosis
ORO: Oil red O
P: phosphorus
qPCR: quantitative PCR
SEM: standard error of the mean
SMI: structure model index
Tb.N: trabecular number
Tb.Sp: trabecular separation
TfR1: transferrin receptor 1

Osteoporosis (OP) is a common progressive, systemic and chronic bone disease that primarily affects older adults and postmenopausal women [1]. With the elderly population increasing rapidly, the incidence rate of OP is growing gradually [2]. According to the statistical analysis, more than 50% of the women, who are older than 50 years, suffer from OP [3]. OP is characterized by microstructure degradation of osseous tissues, decreased bone mineral density (BMD) and increased osteopsathyrosis [4]. OP has increased the financial burden and impaired the health of patients with OP [5]. Many factors, including genetic factors, hormones, cytokines and aging, can induce OP. Among these factors, estrogen deficiency, aging and metabolic disorders are the main causes of OP [6]. However, some studies have indicated that the imbalance of microelement is a new risk factor for OP [7]. According to previous reports, many researchers focus on the relationship between OP and calcium (Ca), phosphorus (P) and magnesium (Mg) [8, 9, 10]. In recent articles, it has been reported that ironmight be related to bone metabolism, and iron loading plays an important role in the development of OP [11, 12].

It is universally recognized that iron is an essential element for many cellular and molecular process, which can participate in multiple biological processes in human bodies, including cell growth, DNA synthesis, oxygen transport, various enzymatic activities, electron transport and responses of immune systems [13, 14, 15]. In normal conditions, iron homeostasis is accurately regulated [16]. On the one hand, iron depletion could influence multiple biological processes in the human body [17]. On the other hand, excess iron is harmful, resulting in cellular dysfunction, organ damage, high mortality rates and bone loss [18]. The imbalance of iron homeostasis, either iron deficiency or iron loading, can result in the dysfunction of various tissues, organs and cells [19]. Increasing evidence has reported that iron loading is related to OP [18]. For example, it has been reported that iron loading could affect the balance of osteoblast metabolism and promote apoptosis *in vitro*, which induce bone loss and OP [20]. Iron loading could suppress the osteoblast‐mediated bone formation, may also improve the osteoclast‐mediated bone resorption and further may play a key role in the development of OP [21]. Furthermore, it has been reported that iron‐loaded patients are at high risk for OP because of the suppressed bone remodeling by iron accumulation [22]. However, very little is known about the therapeutic methods for treating iron loading‐induced OP. Therefore, further exploration of the novel therapeutic strategies for treating iron loading‐induced OP is urgently needed.

Bone marrow mesenchymal stem cells (BMSCs) are important members of the stem cells and regarded as the seed cells in many fields [23]. It has been shown that BMSCs are a type of multipotent stem cells, which can differentiate into various types of cells, including osteoblasts, adipose cells, cartilage cells, muscle cells and nerve cells [24]. Thus, it is broadly discussed that BMSCs are considered as potential cell therapies for diverse medical conditions, such as OP [25]. Numerous studies have shown that iron regulates the biological functions of BMSCs, including osteoblast activity, extracellular matrix mineralization and osteogenic differentiation [26, 27, 28, 29]. Besides, iron loading is closely related to elevated ferritin, and ferritin expression caused by iron loading could inhibit the osteogenic differentiation of BMSCs because of its ferroxidase activity [27].

Besides, astragaloside IV (ASI‐IV), a pure compound derived from Radix Astragali, has been reported to exhibit various bioactivities with no toxicity, such as cardioprotective, anti‐inflammatory, antioxidant, antiasthmatic and anticancer effects [30, 31]. Therefore, ASI‐IV can be used for the treatment of degenerative bone diseases, including periodontal disease, rheumatoid arthritis and aseptic prosthesis loosening [32, 33]. However, its protective effects against dysfunction of BMSCs induced by iron loading have not been investigated.

To the best of our knowledge, the roles of ASI‐IV in BMSCs have not been tested. In this study, BMSCs were treated with iron loading and then treated with ASI‐IV to investigate whether ASI‐IV exerted protective effects against dysfunction of BMSCs caused by iron loading. We thus examined the effects of ASI‐IV on the cell viability, proliferation, pluripotency, osteogenesis and adipogenesis of BMSCs treated with iron loading. Also, ASI‐IV played a protective effect in iron loading‐induced abnormal differentiation of BMSCs by regulation of iron homeostasis and metabolism, which inhibited the iron loading by regulating the expression of ferritin, transferrin receptor 1 (TfR1) and Fpn1, but not divalent metal ion binding protein (DMT1). Our study provided both basic research on stem cell biology and applications of ASI‐IV for treating iron loading‐induced OP.

## Materials and methods

### Cell culture

All procedures were approved by the Ethics Committee of the First Affiliated Hospital of Harbin Medical University. The 8‐week‐old female C57BL/6J mice (about 18 g) were used to separate the tibias and femurs under pathogen‐free conditions according to the previous studies [24, 26, 34]. The bone tissues were treated with 75% alcohol, and the surrounding tissues were removed. F12–Dulbecco’s modified Eagle’s medium (DMEM; HyClone, Logan, UT, USA) was used to flush bone marrow of cavity. After six flushes, the bone marrow and medium were plated in a 25‐cm^2^ cell culture flask (Nest, Wuxi, China). The cells were cultured in F12–DMEM (HyClone) supplemented with 10% FBS (Thermo, Carlsbad, CA, USA) and 1% penicillin–streptomycin (Beyotime, China), and the cells were maintained in a 5% CO_2_ incubator (Thermo, USA) at 37 °C. After 6 h, the medium was replaced by fresh medium, and the nonadherent cells were removed. When cell confluence reached about 90%, the cells were treated with trypsin (Beyotime) and passaged to next passage. By using specific medium and trypsin, BMSCs were obtained. The BMSCs exerted a spindle‐shape morphology, which expressed the surface markers cluster of differentiation 29 (CD29), CD44, CD73, CD90, CD105 and CD166, but not the hematopoietic markers CD45 and CD34, detected by using flow cytometers.

The adipogenic differentiation medium (Cyagen Biosciences, Santa Clara, CA, USA) containing 10% FBS, 1% penicillin–streptomycin, 1% glutamine, 0.1% dexamethasone, 0.2% insulin, 0.1% rosiglitazone and 0.1% isobutylmethylxanthine was applied. The osteogenic differentiation‐induced medium (Cyagen Biosciences) supplemented with 10% FBS, 1% glutamine, 0.2% ascorbic acid, 1% penicillin–streptomycin, 0.01% dexamethasone and 1% β‐glycerophosphate was used for osteogenic differentiation.

### Ferric ammonium citrate treatment

Ferric ammonium citrate (FAC) was applied to construct iron loading BMSC models. BMSCs were respectively treated with FAC at 25, 50, 100, 200 and 400 μm. BMSCs from the control group were treated with PBS.

### ASI‐IV treatment

ASI‐IV was used to determine whether ASI‐IV can exhibit the protective roles in FAC‐treated BMSCs. BMSCs were respectively treated with ASI‐IV at 25, 50, 100 and 200 μm.

### Cell Counting Kit‐8 assay

Cell Counting Kit‐8 (CCK‐8) assay was used to detect the cell viability of BMSCs according to the manufacturer’s instructions of the CCK‐8 detection kit (Tongren, Tokyo, Japan). In brief, BMSCs were seeded into a 96‐well plate (Nest) and cultured in normal growth medium. After 24 h, the cells were treated with different doses of FAC or ASI‐IV for 24 h. Then 10 μL CCK‐8 solution and 100 μL F12–DMEM without 10% FBS or 1% penicillin–streptomycin were added into each well. Then the cells were incubated for 2 h at 37 °C temperature in a dark condition. Finally, the absorbance at 450 nm (*A*_450 nm_) was assessed using a microplate reader (TECAN, Männedorf, Switzerland).

### Colony formation assay

Cell colony formation assay was performed to detect the proliferation ability of BMSCs. First, BMSCs in single‐cell suspension were seeded into six‐well plates (Nest) at a density of 100 per well and cultured in normal growth medium. The cells were cultured for 2 weeks, and the medium was changed every 3 days. Then the colonies were washed by PBS and fixed in 4% PFA solution, and the cells were incubated in 0.4% crystal violet (Biosharp, Hefei, China) for 20 min. Finally, the number of colonies was observed and calculated under an inverted light microscope (Olympus, Hachioji, Japan).

### Real‐time quantitative PCR analysis

Real‐time quantitative PCR (qPCR) was applied to quantify the expression of target genes. Total RNAs were extracted by using TRIzol reagents (Thermo, USA) following the manufacturer's instructions. cDNAs were obtained by using cDNA Synthesis Kit (ABI, Carlsbad, CA, USA). Then the cDNAs were amplified by SYBR Green reagents (Vazyme, Nanjing, China) by using an Applied Biosystems 7500 Real‐Time PCR system (ABI), and the cycling conditions were as follows: 30 s of polymerase activation at 95 °C, followed by 40 cycles of 95 °C for 5 s and 60 °C for 30 s. The primers were designed and synthesized by GenePharma, Shanghai, China. The primers used in this study are shown in Table 1.

**Table 1:** The sequence of primers for real‐time qPCR analysis. F, forward; R, reverse.

Primers	Sequence (5′–3′)
Nanog	F: TCTTCCTGGTCCCCACAGTTT
	R: GCAAGAATAGTTCTCGGGATGAA
Sox2	F: GCGGAGTGGAAACTTTTGTCC
	R: CGGGAAGCGTGTACTTATCCTT
OCT4	F: GGCTTCAGACTTCGCCTCC
	R: AACCTGAGGTCCACAGTATGC
ALP	F: GTTGCCAAGCTGGGAAGAACAC
	R: CCCACCCCGCTATTCCAAAC
OCN	F: GGGAGACAACAGGGAGGAAAC
	R: CAGGCTTCCTGCCAGTACCT
PPARγ	F: GGAAAGACA ACGGACAAATCA
	R: TACGGATCGAAACTGGCAA
FABP4	F: ACACTGGTCCTAGCTGTATTCT
	R: CCAGCCACGTTGCATTGTA
GAPDH	F: GACAAAATGGTGAAGGTCGGT
	R: GAGGTCAATGAAGGGGTCG

### Alizarin red S staining

BMSCs were plated in a 24‐well plate (Nest) and cultured in normal growth medium. When the cells grew to about 80%, the medium was replaced with osteogenic induction medium (Cyagen, Guangzhou, China) containing 10% FBS, 1% glutamine, 1% penicillin–streptomycin, 0.2% ascorbic acid, 1% β‐glycerophosphate and 0.01% dexamethasone. After 21 days, the osteogenic differentiation was assessed by performing Alizarin red S (ARS) staining. First, a 500 μL volume of ARS staining reagent (Cyagen, China) was added into the cells. After incubation for 30 min, the solution was discarded and replaced by PBS. Finally, 10 pictures were taken randomly by an inverted light microscope.

### Oil red O staining

Oil red O (ORO) staining was performed to confirm the adipogenic differentiation of BMSCs. BMSCs were plated in a 24‐well plate and cultured in normal growth medium. When the cells grew to about 90%, the medium was replaced by adipogenic induction medium (Cyagen, China). After 24 days, the cells were washed twice with PBS and fixed with 4% paraformaldehyde (PFA) for 30 min at room temperature. Then the cells were stained with ORO staining solution for 30 min and observed under a light microscope.

### Animals and construction of osteoporotic mice

Female‐specific pathogen‐free C57BL/6J mice (18 g, 8 weeks old) were provided by the Laboratory Animal Center of Second Affiliated Hospital of Harbin Medical University. The mice were kept in hygienic plastic cages in a clean well‐ventilated room and were given free access to food and water with normal light and dark cycles. The mice were given free access to water and food. Thirty‐six mice were randomly assigned to three groups: sham group (*n* = 12), iron loading group (*n* = 12) and iron loading + ASI‐IV treatment group (*n* = 12). Besides, 250 mg·kg^−1^ iron dextran (Fe‐dextran; Sigma‐Aldrich, St. Louis, MO, USA) was injected intraperitoneally into the mice from the iron loading group every other day for 4 weeks. The same volume of PBS was injected intraperitoneally into the mice from the sham group. ASI‐IV at a dose of 40 mg·kg^−1^ was injected intraperitoneally into the mice from the iron loading + ASI‐IV treatment group every other day for 4 weeks. Then the femurs of mice were collected for micro‐computed tomography (micro‐CT) analysis [35]. BMSCs were isolated according to the previous study and used for additional analysis. The study was approved by the Medical Research Animal Ethics Committee of The First Affiliated Hospital of Harbin Medical University.

### Detection of serum iron

Serum iron content of mice was detected by Serum Iron Concentration Kit (BC1730; Solarbio, Peking, China) using spectrophotometric assay. All the procedures were performed according to the instructions of Serum Iron Kit.

### Micro‐CT analysis

All examinations were conducted according to the principles and procedures of the *Care and Use of Laboratory Animals* of the First Affiliated Hospital of Harbin Medical University. The right femurs of the mice were collected, and analysis of the trabecular microarchitecture was obtained by micro‐CT machine (Scanco, Brüttisellen, Switzerland) at 70 KVp, 114 μA for 800 ms. Bone morphometric parameters, including the trabecular number (Tb.N), trabecular separation (Tb.Sp), structure model index (SMI) and BMD, were acquired from the micro‐CT analysis.

### Western blot analysis

Total proteins were extracted by using lysis buffer (Beyotime), separated by SDS/PAGE gels and transferred to the membranes (Millipore, MA, USA). After hybridization, the membranes were incubated with the primary antibody at 4 °C condition for one night. In this study, the primary antibodies used were: β‐actin (IgG, CST, USA), ferritin (IgG, Abcam, Cambridge, Britain), ferritin (IgG, Abcam), DMT1 (IgG, Abcam), Fpn1 (IgG, Abcam) and TfR1 (IgG, Abcam). The next day, the membranes were immunoblotted with a secondary antibody (CST, Danvers, MA, USA) at room temperature for 60 min. The intensity of protein bands was analyzed using imagej software (NIH, USA).

### Statistical analysis

All numerical data in this study were presented as mean ± standard error of the mean (SEM). Differences in numerical data between two groups were determined by using the unpaired Student's *t*‐test. ANOVA followed by Tukey’s *post hoc* analysis was performed for comparisons of multiple groups. The analysis was performed by using the graphpad prism software (GraphPad Software, San Diego, CA, USA). A *P* value <0.05 was considered as statistically significant.

## Results

### Iron loading suppressed the cell viability, proliferation and pluripotency of BMSCs

**Fig. 1:**
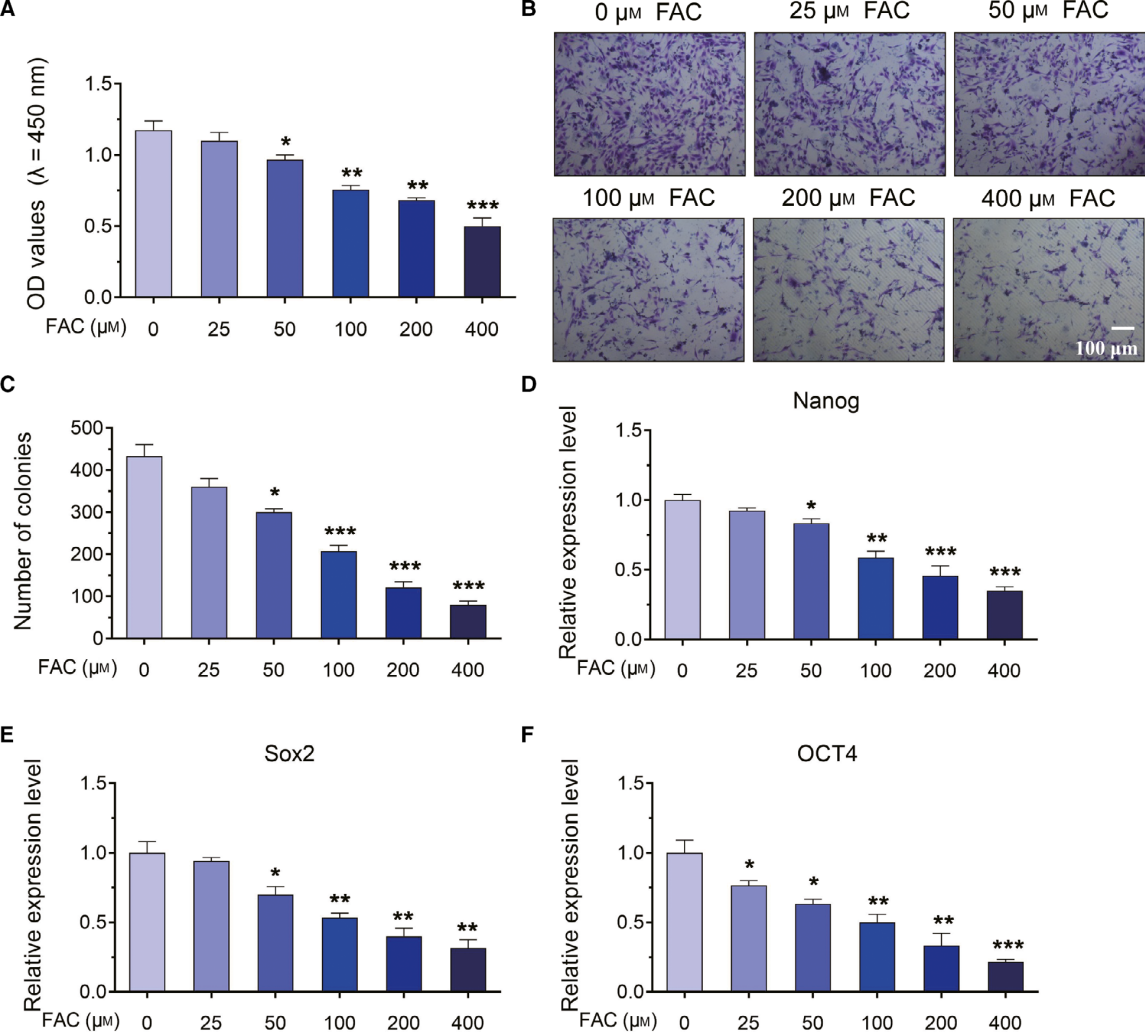
The effects of iron loading on the cell viability, proliferation and pluripotency of BMSCs. (A) BMSCs were treated with various concentrations of FAC for 24 h. CCK‐8 was used to detect the cell viability of BMSCs. (B) Colony formation assay was used to examine the proliferation ability of BMSCs. Scale bar, 100 μm. (C) Statistical results of colony formation assay. (D–F) Real‐time qPCR was performed to assess the expression level of pluripotency‐related genes. Values represent the mean ± SEM of at least three independent experiments. ANOVA followed by Tukey’s *post hoc* analysis was used. *P < 0.05, **P < 0.01, ***P < 0.001, as compared with control.

To investigate the roles of iron loading in the biological functions of BMSCs, we applied FAC to simulate iron loading conditions. BMSCs were treated with FAC at a final concentration of iron at 25, 50, 100, 200 or 400 μm. The BMSCs without FAC treatment served as the control group. After FAC treatment of 24 h, CCK‐8 assay was performed to detect the cell viability of different groups of BMSCs. As shown in Fig. 1A, the cell viability was gradually decreased, along with the concentration of FAC being increased (Fig. 1A). Then colony formation assay was used to examine the proliferation ability of BMSCs after FAC treatment. The results of colony formation assay indicated that, as the concentration of FAC increased, the number of colonies was gradually reduced, showing that the proliferation ability of BMSCs was decreased under iron loading conditions (Fig. 1B,C). Besides, to test the pluripotency of BMSCs, we used real‐time qPCR analysis to detect the relative expression of pluripotency‐related genes, including Nanog, Sox2 and OCT4. The results of real‐time qPCR analysis revealed that FAC dramatically inhibited the expression of Nanog, Sox2 and OCT4 in a dose‐dependent manner (Fig. 1D–F). Therefore, these results suggested that iron loading induced by FAC significantly inhibited the cell viability, proliferation and pluripotency of BMSCs.

### Iron loading inhibited the osteogenic differentiation and promoted adipogenic differentiation of BMSCs

**Fig. 2:**
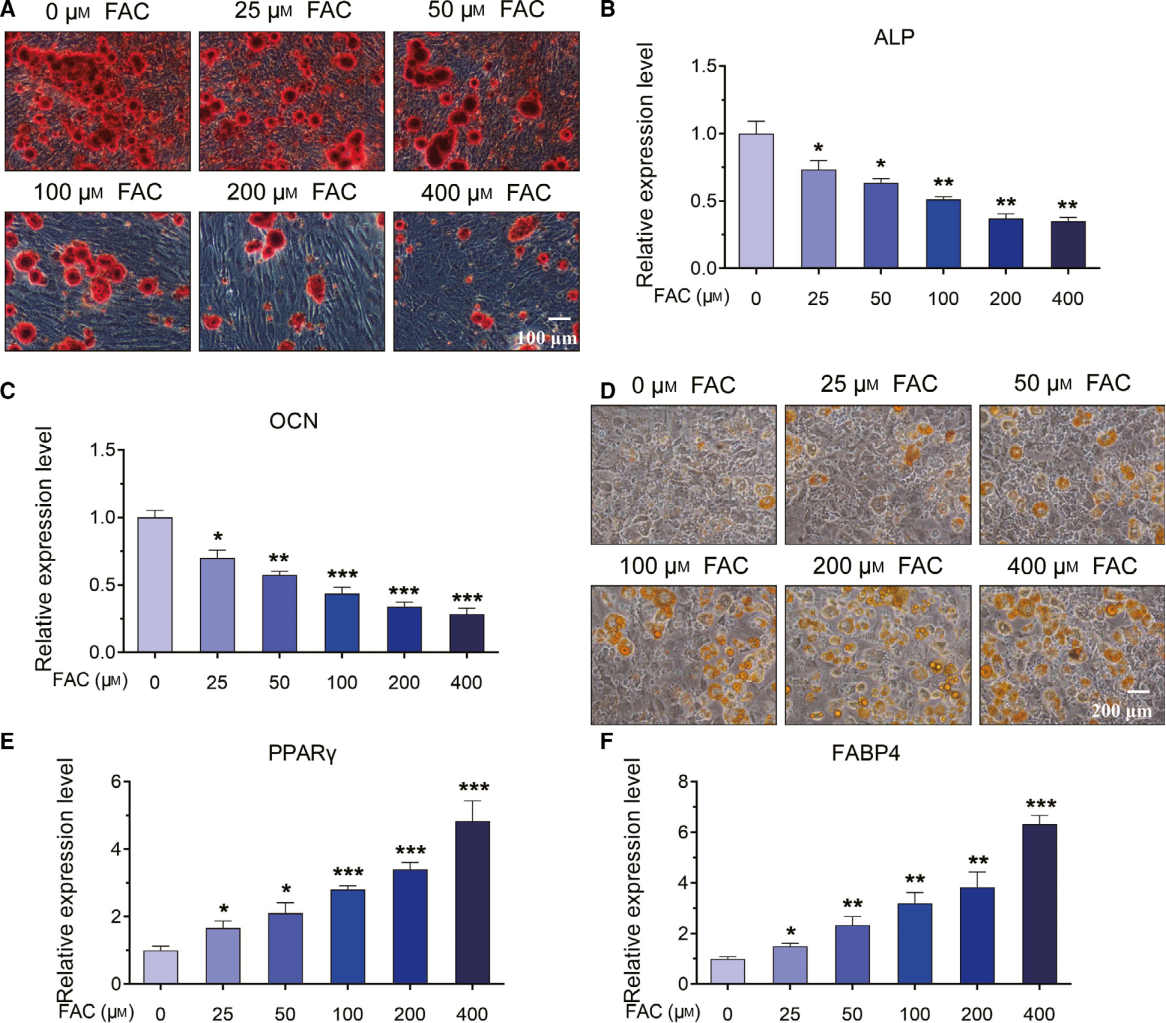
The roles of iron loading in the osteogenesis and adipogenesis of BMSCs. (A) BMSCs were treated with various concentrations of FAC for 24 h, and the cells were cultured in osteogenic induction medium for 21 days. ARS staining was applied to explore the osteogenic differentiation of BMSCs. Scale bar, 100 μm. (B, C) Real‐time qPCR was performed to assess the expression level of osteogenesis‐related genes, including ALP and OCN. (D) BMSCs were treated with various concentrations of FAC for 24 h, and the cells were cultured in adipogenic induction medium for 24 days. ORO staining was used to monitor the adipogenic differentiation of BMSCs. Scale bar, 200 μm. (E, F) Real‐time qPCR was performed to assess the expression level of adipogenesis‐related genes, including PPARγ and FABP4. Values represent the mean ± SEM of at least three independent experiments. ANOVA followed by Tukey’s *post hoc* analysis was used. *P < 0.05, **P < 0.01, ***P < 0.001, as compared with control.

To investigate the effects of iron loading on the differentiation of BMSCs, we treated BMSCs with different concentrations of FAC for 24 h. Then the BMSCs were cultured in osteogenic induction medium. After 21 days, ARS staining was performed to detect the osteogenesis of BMSCs. As shown in Fig. 2A, as the concentration of FAC increased, the formation of mineralized nodules gradually decreased under iron loading conditions, which suggested that the osteogenic differentiation was damaged by iron loading (Fig. 2A). Besides, the expression level of osteogenesis‐related genes, including ALP and OCN, was detected by real‐time qPCR analysis. The results uncovered that the expression of ALP and OCN was observably down‐regulated after treatment of FAC, suggesting that iron loading significantly inhibited the osteogenic differentiation of BMSCs (Fig. 2B,C).

Furthermore, the roles of FAC in the adipogenic differentiation were verified after the BMSCs were treated with adipogenic induction medium for 24 days. ORO staining showed that different concentrations of FAC could increase the number of oil droplets, indicating that FAC promoted the adipogenic differentiation of BMSCs (Fig. 2D). We also observed that FAC significantly increased the expression of adipogenesis‐related genes, including PPARγ and FABP4, in a dose‐dependent manner (Fig. 2E,F). Thus, the earlier results indicated that iron loading induced by FAC could suppress the osteogenesis, but accelerate the adipogenesis, of BMSCs.

### ASI‐IV neutralized the effect of iron loading on the cell viability, proliferation and pluripotency of BMSCs

**Fig. 3:**
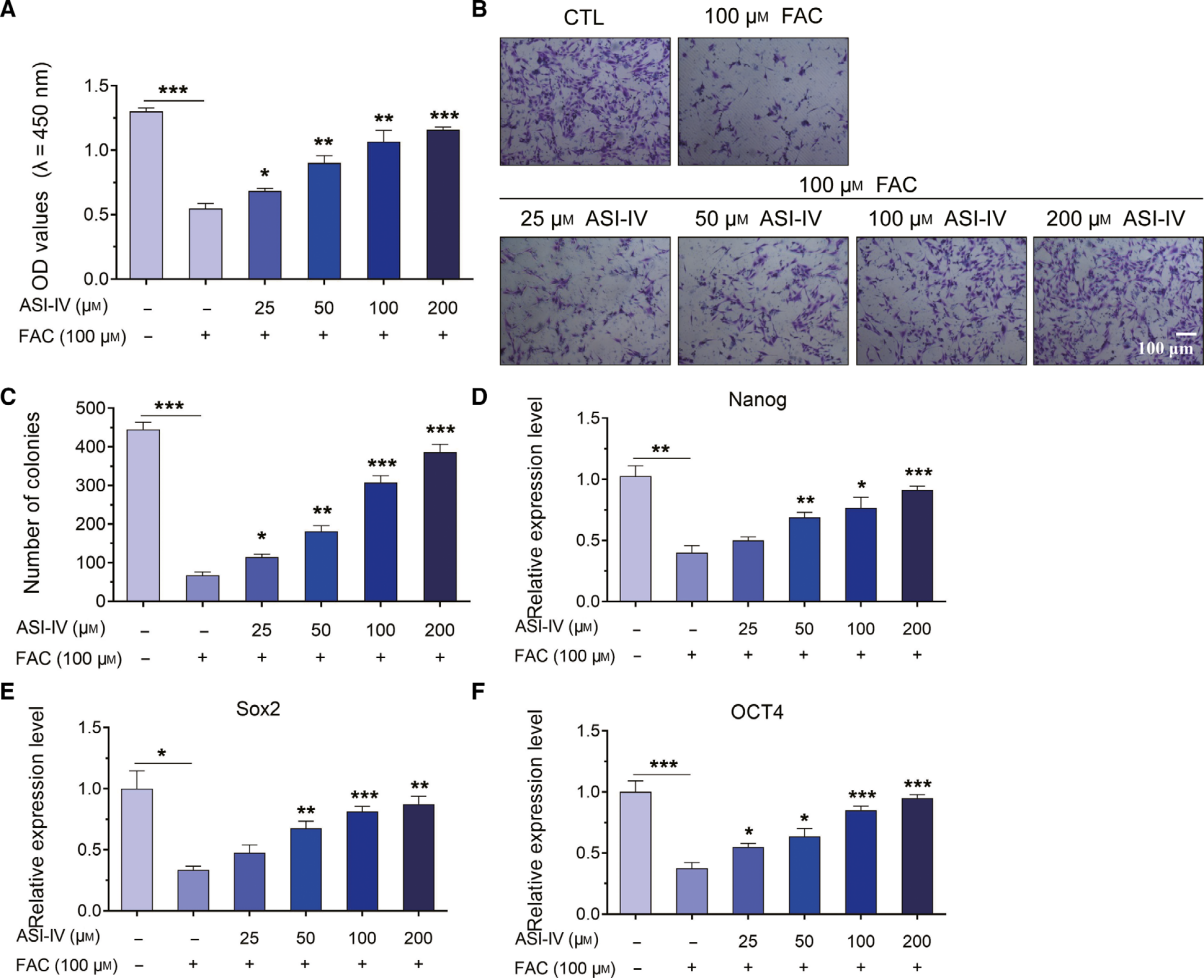
ASI‐IV treatment inhibited iron loading‐induced injury of cell viability, proliferation and pluripotency in BMSCs. (A) BMSCs cells were treated with 100 μm FAC and increasing concentrations of ASI‐IV (25, 50, 100 and 200 μm) for 24 h, respectively. Cell viability was determined using CCK‐8 assay. (B, C) BMSCs were treated with 100 μm FAC, with or without various concentrations of ASI‐IV (25, 50, 100 or 200 μm) for 24 h. Cell proliferation was examined by using colony formation assay. Scale bar, 100 μm. (D–F) BMSCs were treated with different concentrations of ASI‐IV (25, 50, 100 or 200 μm) under iron loading conditions for 24 h. Values represent the mean ± SEM of at least three independent experiments. ANOVA followed by Tukey’s *post hoc* analysis was used. *P < 0.05, **P < 0.01, ***P < 0.001, as compared with control.

We have verified that iron loading exhibited cytotoxic effects on the biological functions of BMSCs. Thus, we continued to explore a novel and potential drug that could play a protective role in the BMSCs injury induced by iron loading. ASI‐IV has been reported to play a positive role in the biological functions of osteoblasts [32, 36, 37]. Therefore, ASI‐IV might be associated with the biological functions of BMSCs and may exhibit protective roles in the dysfunction of BMSCs under iron loading condition. In this study, among different concentrations at 25, 50, 100, 200 or 400 μm, we chose FAC at 100 μm as the optimum concentration in view of cytotoxicity. To explore the effect of ASI‐IV on BMSCs after treatment of FAC, we treated BMSCs with FAC at 100 μm and with ASI‐IV at different concentrations, including 25, 50, 100 or 200 μm. As shown in Fig. 3A, exposure to FAC induced a significant decrease in the cell viability of BMSCs (Fig. 3A). Conversely, ASI‐IV treatment strongly increased the cell viability, which was inhibited under iron loading conditions, in a dose‐dependent manner (Fig. 3A). Besides, exposure to 100 μm FAC significantly reduced the formation of colonies, which could be reversed by different concentrations of ASI‐IV at 25, 50, 100 or 200 μm (Fig. 3B,C). The results revealed that ASI‐IV at 25, 50, 100 or 200 μm was able to remove the inhibition of FAC on the proliferation ability of BMSCs. In addition, real‐time qPCR analysis showed that the decreased expression level of Nanog, Sox2 and OCT4 caused by 100 μm FAC was elevated by ASI‐IV treatment in a dose‐dependent manner, indicating that ASI‐IV treatment also reduced the toxic effects of FAC on the pluripotency of BMSCs (Fig. 3D–F). These findings demonstrated that FAC‐induced injury on the cell viability, proliferation and pluripotency was significantly improved by ASI‐IV treatment.

### The effects of FAC on the osteogenesis and adipogenesis were reversed by ASI‐IV treatment

**Fig. 4:**
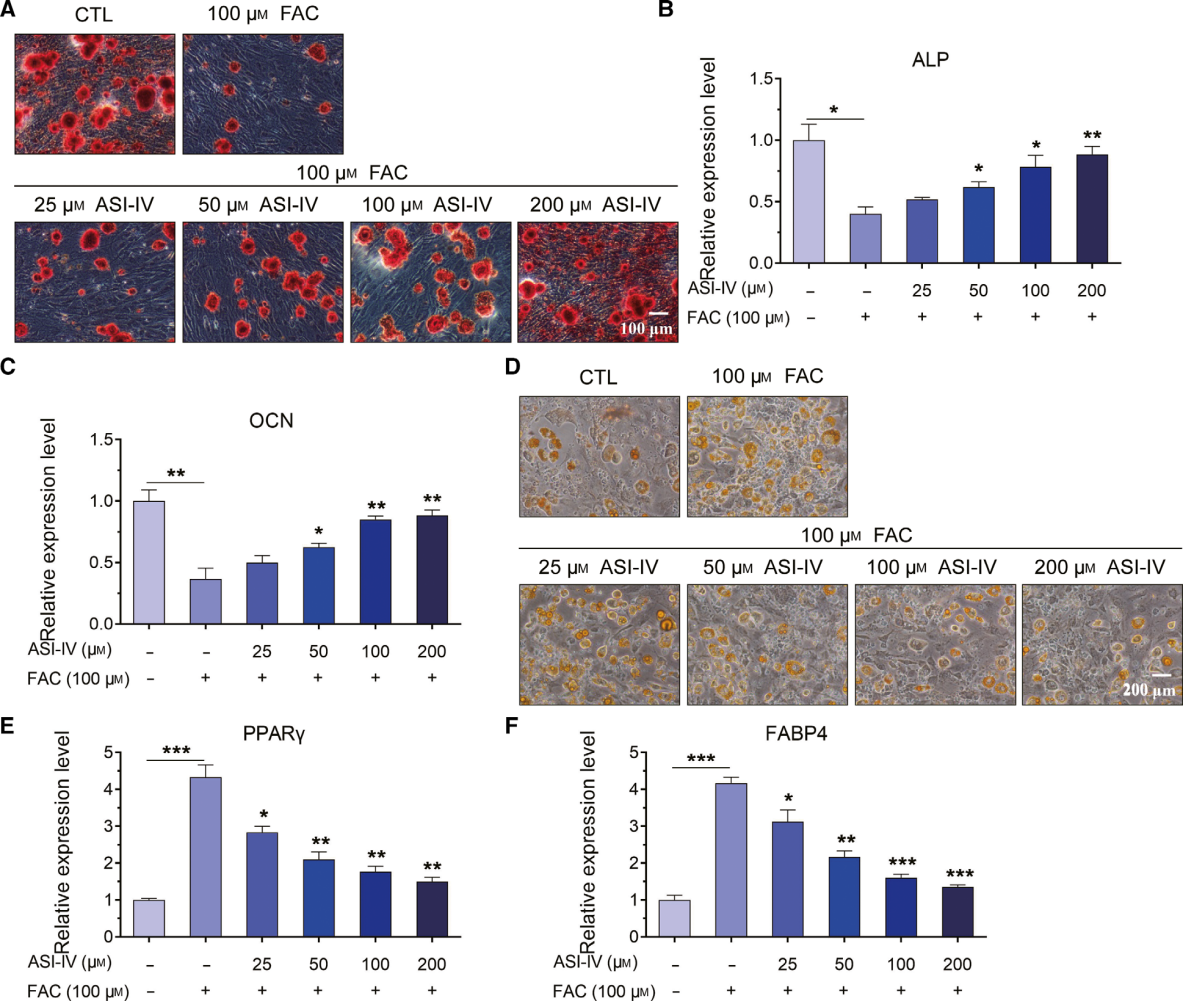
ASI‐IV reversed the iron‐induced reduced osteogenesis and enhanced adipogenesis of BMSCs. (A) Representative microscopy photomicrographs of ARS staining of BMSCs after treatment with FAC and ASI‐IV. Scale bar, 100 μm. (B, C) ASI‐IV elevated the mRNA expression of ALP and OCN in BMSCs under iron loading conditions. (D) Representative graphs of ORO staining of BMSCs after treatment with FAC and ASI‐IV. Scale bar, 200 μm. (E, F) The relative expression level of PPARγ and FABP4 was quantified in BMSCs treated with 100 μm FAC, with or without various concentrations of ASI‐IV (25, 50, 100 or 200 μm) for 24 h. Values represent the mean ± SEM of at least three independent experiments. ANOVA followed by Tukey’s *post hoc* analysis was used. *P < 0.05, **P < 0.01, ***P < 0.001, as compared with control.

To further study the role of ASI‐IV in the osteogenesis and adipogenesis of BMSCs subjected to iron loading, we treated BMSCs with 100 μm FAC and 25, 50, 100 or 200 μm ASI‐IV. The BMSCs were induced into osteoblasts for 21 days or adipocytes for 24 days. As the concentration of ASI‐IV increased, the amount of matrix mineralization was gradually up‐regulated under iron loading conditions (Fig. 4A). In addition, real‐time qPCR assay revealed that iron loading significantly decreased the mRNA expression of osteogenesis‐related genes, including ALP and OCN (Fig. 4B,C). Interestingly, the mRNA expression of ALP and OCN was significantly up‐regulated in FAC‐ and ASI‐IV‐treated BMSCs, compared with that of FAC‐treated cells (Fig. 4B,C). Therefore, ASI‐IV treatment protected BMSCs from the deleterious effects of iron loading on the osteoblastic differentiation.

Furthermore, ORO staining suggested that the adipogenic differentiation showed a steady and slight decrease after the exposure to ASI‐IV depending on the concentrations when treated with FAC at a concentration of 100 μm (Fig. 4D). We compared the adipogenesis‐related genes of BMSCs after treatment with FAC and FAC + ASI‐IV by performing real‐time qPCR analysis (Fig. 4E,F). The results of real‐time qPCR analysis showed that BMSCs treated with different concentrations of ASI‐IV showed lower expression levels of PPARγ and FABP4 than those in the FAC‐treated groups (Fig. 4E,F). These results indicated that ASI‐IV protected BMSCs from iron loading‐induced decreased osteogenesis but inhibited the adipogenesis caused by iron loading.

### ASI‐IV exerted protective effects on iron loading‐induced osteoporotic mice

**Fig. 5:**
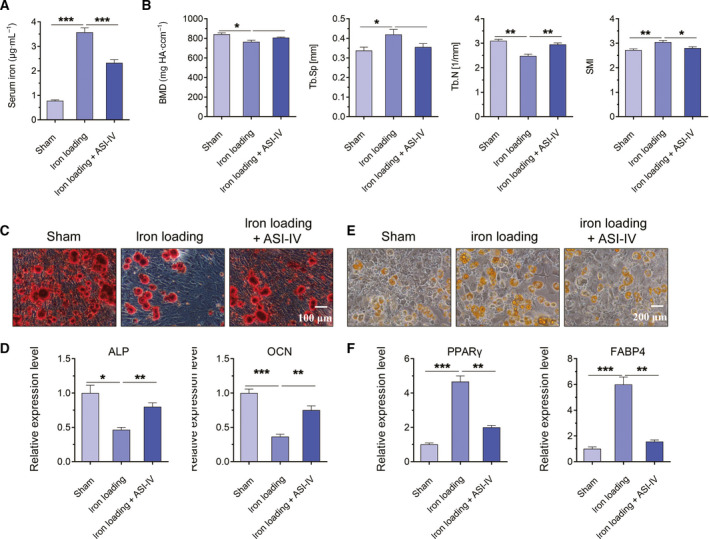
ASI‐IV attenuated iron loading‐induced bone loss *in vivo*. (A) The serum iron of mice from the sham and iron loading groups was analyzed by spectrophotometric assay. (B) Representative data of micro‐CT analysis of femurs from the sham, iron loading and iron loading + ASI‐IV groups. Decreased BMD and Tb.N and increased Tb.Sp and SMI. (C) Representative photomicrographs of ARS staining in BMSCs with or without ASI‐IV under iron loading conditions. Scale bar, 100 μm. (D) ASI‐IV exerted protective effects on iron loading‐induced osteogenic differentiation. (E) Representative photomicrographs of ORO staining in BMSCs with or without ASI‐IV under iron loading conditions. Scale bar, 200 μm. (F) The mRNA expression of PPARγ and FABP4 was analyzed using real‐time qPCR analysis. Values represent the mean ± SEM of at least three independent experiments. ANOVA followed by Tukey’s *post hoc* analysis was used. *P < 0.05, **P < 0.01, ***P < 0.001, as compared with the sham group.

To further examine the effect of ASI‐IV on iron loading‐induced OP *in vivo*, we randomly assigned the mice to three groups: sham group, iron loading group and iron loading + ASI‐IV treatment group. The mice were administrated with 250 mg·kg^−1^ Fe‐dextran intraperitoneally, with or without ASI‐IV (40 mg·kg^−1^) for 4 weeks. To explore whether the iron loading condition was successfully established, we analyzed the level of serum iron from sham, iron loading and iron loading + ASI‐IV groups using spectrophotometric assay. The results indicated that the level of serum iron was markedly increased in the mice from the iron loading group, suggesting that there was iron accumulation in mice (Fig. 5A). Furthermore, the level of serum iron was decreased in the mice from the iron loading + ASI‐IV group, indicating that ASI‐IV had an effect on iron homeostasis (Fig. 5A). Then the femurs were collected for micro‐CT analysis to detect the trabecular bone changes of different model groups. As shown in Fig. 5B, the iron loading group exhibited a significant trabecular bone loss, as revealed by decreased BMD and Tb.N and increased Tb.Sp and SMI, compared with the sham group. ASI‐IV treatment improved BMD and Tb.N and suppressed the degeneration of trabecular bone in mice treated with Fe‐dextran (Fig. 5B). Besides, the BMSCs were isolated from the sham, iron loading and iron loading + ASI‐IV treatment groups. After osteogenic differentiation for 21 days, ARS staining was performed to assess the osteogenesis of BMSCs. As shown in Fig. 5C, iron loading reduced the mineralization process of BMSCs in the iron loading group, and ASI‐IV administration nearly restored it to the normal level (Fig. 5C). The significant decrease was observed in ALP and OCN level in BMSCs from the iron loading group, whereas ALP and OCN level was markedly increased by ASI‐IV treatment (Fig. 5D). Altogether, these results revealed that ASI‐IV could elevate the bone density and inhibit trabecular bone loss induced by iron loading.

Then, to further explore the effects of ASI‐IV on iron loading‐induced adipocyte formation, we isolated and induced BMSCs into adipocytes for 24 days. As shown in Fig. 5E, the area and number of adipocytes increased in the iron loading groups but decreased after treatment of ASI‐IV (Fig. 5E). Moreover, with the administration of iron loading, greater expression of PPARγ and FABP4 was discovered in BMSCs (Fig. 5F), and the increased expression levels of PPARγ and FABP4 in the iron loading condition were reversed by ASI‐IV treatment (Fig. 5F). The above results suggested that ASI‐IV could exert protective effects on iron loading‐induced osteoporotic mice.

### ASI‐IV played a protective effect in iron loading‐induced abnormal differentiation of BMSCs by regulating the iron homeostasis and metabolism

**Fig. 6:**
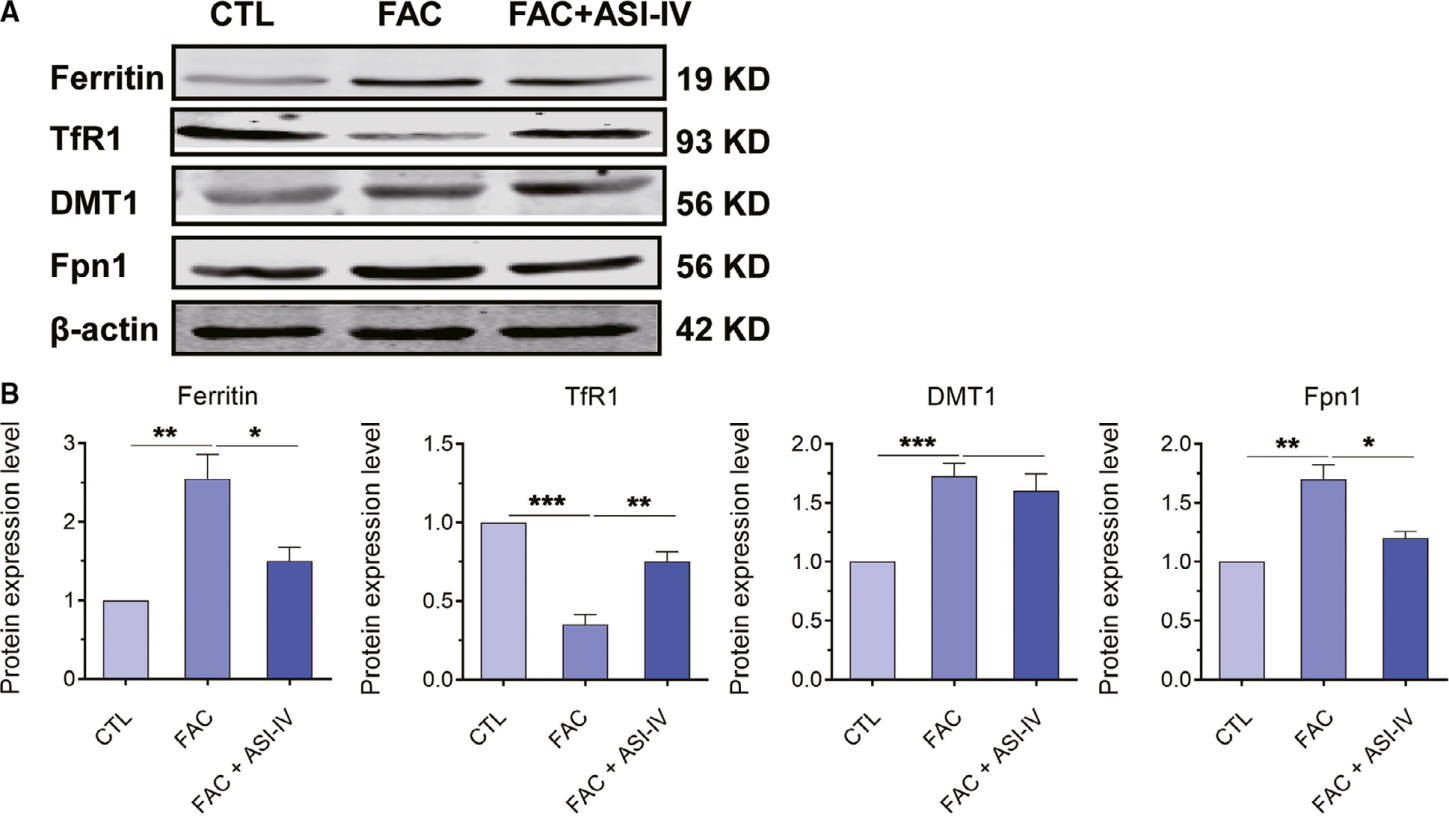
Effects of ASI‐IV on expression of ferritin, TfR1, Fpn1 and DMT1 in BMSCs pretreated with FAC. (A) BMSCs were treated with 100 μm FAC and 200 μm ASI‐IV for 24 h, and then western blot analysis was performed to detect the protein expression of ferritin, TfR1, Fpn1 and DMT1. (B) Quantitative analysis of western blot analysis. Values represent the mean ± SEM of at least three independent experiments. ANOVA followed by Tukey’s *post hoc* analysis was used. *P < 0.05, **P < 0.01, ***P < 0.001, as compared with controls.

To explore whether ASI‐IV played a protective effect in iron loading‐induced abnormal differentiation of BMSCs via regulation of the iron homeostasis and iron metabolism, we treated BMSCs with 100 μm FAC and 200 μm ASI‐IV for 24 h. Then the protein levels of iron storage protein ferritin, iron import protein TfR1 and DMT1, and export protein ferroportin 1 (Fpn1) were detected by western blot analysis. As shown in Fig. 6A,B, 100 μm FAC markedly elevated the expression of ferritin, DMT1 and Fpn1 and reduced the expression of TfR1, which was partially blocked by 200 μm ASI‐IV treatment. Furthermore, the increased expression of DMT1 caused by FAC treatment was not changed by 200 μm ASI‐IV (Fig. 6A,B). The earlier results suggested that ASI‐IV played a protective effect in iron loading‐induced abnormal differentiation of BMSCs by regulation of iron homeostasis and metabolism, which inhibited the iron loading by regulating the expression of ferritin, TfR1 and Fpn1, but not DMT1.

## Discussion

As the previous reports showed, BMSCs are a type of stem cell with specialization potentials, which can be differentiated into various types of cells, including osteoblasts, adipocytes, chondrocytes, myocytes and neurocytes [38]. In bone microenvironment, osteoblast cells are mainly derived from BMSCs, and osteoblasts are responsible for bone development [39]. Therefore, the biological functions of BMSCs are vital for the homeostasis of bone. Iron is an important nutritional element for the human body [40]. However, excess iron accumulation could bring damage to cells, tissues and even organs, leading to various types of disease [41]. It has been discovered that iron loading is correlated with dysfunction in bone metabolism, including OP [42].

This study investigated the effects of ASI‐IV treatment on the biological functions of BMSCs, including cell viability, proliferation, pluripotency, osteogenesis and adipogenesis. Different concentrations of FAC (25, 50, 100, 200, 400 μm) suppressed the BMSC cell viability, proliferation, pluripotency and osteogenic differentiation in a dose‐dependent manner, which was lowest at 400 μm, and FAC at 25, 50, 100, 200 or 400 μm stimulated the adipogenic differentiation of BMSCs in a dose‐dependent manner, which peaked at 400 μm. This result was consistent with that described in a previous report, which showed that FAC treatment induced the dysfunction of BMSCs [26]. Moreover, FAC treatment at a concentration of 100 μm significantly and consistently exhibited the effective role, and it did not bring much damage to the BMSCs. This result indicated that the FAC at 100 μm was the optimum concentration, in accordance with a previous study indicating FAC at 100 μm as the optimal treatment dose [29]. Also, it was different from the present study that FAC at 200 μm was chosen as the optimal concentration in a previous study [27]. However, to the best of our knowledge, no previous reports have explored the role of ASI‐IV in the biological functions of BMSCs, including cell viability, proliferation, pluripotency, osteogenesis and adipogenesis of BMSCs under FAC‐induced iron loading conditions.

ARS staining showed that treatment with ASI‐IV at different concentrations, including 25, 50, 100 or 200 μm, significantly increased the cell viability, proliferation, pluripotency and osteogenesis of BMSCs after exposure to 100 μm FAC, suggesting that ASI‐IV neutralized the effect of iron loading on the cell viability, proliferation, pluripotency and osteogenesis of BMSCs. Adipogenic differentiation of BMSCs is another important factor that can induce OP [43]. Moreover, the effect of ASI‐IV on inhibiting BMSC adipogenic differentiation was further verified by ORO staining and decreased mRNA expressions of PPARγ and FABP4, which are important in the adipogenesis. Moreover, further analysis *in vivo* showed that ASI‐IV exerted protective effects on iron loading‐induced osteoporotic mice. This *in vitro* and *in vivo* study may shed light on the effect of ASI‐IV in rescuing iron loading‐induced OP in mice. Therefore, ASI‐IV might be used as a promising therapeutic agent against iron loading‐induced OP. Until now, there were no reports on iron‐chelating properties of ASI‐IV *in vitro*. Our further analysis explored that ASI‐IV played a protective effect in iron loading‐induced abnormal differentiation of BMSCs by regulation of iron homeostasis and metabolism, which inhibited the iron loading by regulating the expression of ferritin, TfR1 and Fpn1, but not DMT1. Interestingly, our results showed that the expression of ferritin, TfR1 and Fpn1 in iron loading‐induced BMSCs was effectively reduced by ASI‐IV treatment. Therefore, we considered that ASI‐IV might act as an iron chelator to treat iron loading‐mediated damage in BMSCs, and the results suggested that ASI‐IV might be an effective iron chelator to prevent iron overload‐induced injury in other cell types and for use in the treatment of iron loading‐related diseases.

In this study, the antiosteoporotic activity of ASI‐IV was basically analyzed both *in vitro* and *in vivo*. Identification of biological functions observed that ASI‐IV could elevate the cell viability, proliferation, pluripotency and osteogenic differentiation in a dose‐dependent manner and suppress the adipogenic differentiation of BMSCs under iron loading conditions. It was observed by micro‐CT analysis that ASI‐IV can inhibit the bone loss in iron loading‐induced osteoporotic mice. After treatment with ASI‐IV, the osteogenic differentiation of BMSCs from the osteoporotic mice was increased. In contrast, ASI‐IV had an inhibitory effect in the adipogenic differentiation of BMSCs isolated from osteoporotic mice induced by iron loading. Our study for the first time demonstrated that ASI‐IV exerted a positive role in the biological functions of BMSCs under iron loading condition by regulation of iron homeostasis and metabolism. Based on all these results, it was preliminarily concluded that ASI‐IV might serve as a potential candidate for treating iron loading‐induced OP.

## Conflict of interest

The authors declare no conflict of interest.

## Author contributions

WW and JD designed the project and wrote and revised the manuscript. HJ and JD wrote the manuscript and performed sample collection, RNA isolation, real‐time qPCR analysis, ORO staining and ARS staining. HR performed colony formation assay and real‐time qPCR analysis. HJ, JD and GY collected the data and performed the statistical analysis.

## Data Availability

All data will be available from the corresponding author upon reasonable request.
